# Development of an automated transformer-based text analysis framework for monitoring fire door defects in buildings

**DOI:** 10.1038/s41598-025-27648-9

**Published:** 2025-12-16

**Authors:** Seunghyeon Wang

**Affiliations:** Institute for Environmental Design and Engineering, University College London, London, WC1H 0NN UK

**Keywords:** Defective fire door, Construction management, Fire protection, text-based analysis, Deep learning, Bidirectional encoder representations from transformers, Engineering, Mathematics and computing

## Abstract

Fire door defects in residential buildings negatively impact construction management by reducing fire safety effectiveness, increasing the likelihood of smoke and fire spreading, and consequently putting occupant safety at greater risk. To address this critical safety issue, this study proposes and evaluates five transformer-based text classification methods—BERT, RoBERTa, ALBERT, DistilBERT, and XLNet—for automated defect detection. These methods are optimized using both common and method-specific hyperparameters, resulting in 1,458 model variants evaluated through multiple metrics. Among these, the optimized RoBERTa achieves the highest performance, demonstrating F1 scores of 92.13% (frame gap), 87.29% (door closer adjustment), 78.17% (contamination), 82.89% (dent), 80.17% (scratch), 96.66% (sealing components), 98.43% (mechanical components), and 67.81% (others), yielding an average F1 score of 85.44%. Furthermore, RoBERTa significantly outperforms the other optimized 535 text classification models (ANN, SVM, DT, LR, 1D CNN, and LSTM). These results underscore the potential and effectiveness of transformer-based methods for safety management in real-world construction scenarios.

## Introduction

 In residential buildings, fire doors are typically installed at stairwell entrances and front doors of individual units to ensure residents have safe evacuation routes during fires^1^. Additionally, fire doors are strategically engineered to compartmentalize buildings, effectively limiting the spread of fire and smoke between different Sects^2–4^. However, various factors during construction can introduce defects in fire doors. Physical damage often occurs when doors serve as access points during material transportation or interior finishing processes. Furthermore, cost-saving practices, such as using lower-quality materials or employing insufficiently trained workers who do not strictly follow installation guidelines, can significantly undermine fire door integrity^5^.

To verify their proper operation, fire doors undergo visual inspections before the completion of construction. Inspection findings are documented as unstructured textual records, which include details like inspection dates, responsible contractors, and defect locations. Defects recorded are classified into categories, including operational issues (such as doors failing to close properly) and physical damages (such as dents or scratches). This classification is crucial for maintenance teams, enabling them to prioritize safety-critical repairs and improve efficiency by grouping similar repair tasks to minimize downtime and travel between units^6^.

Nevertheless, manually classifying fire door defects from extensive textual descriptions is labor-intensive, time-consuming, and susceptible to errors. Previous research indicates low accuracy in manual defect classification at many construction sites, underscoring the need for automated classification solutions. Earlier studies in the construction field have frequently utilized conventional text classification techniques like Naive Bayes (NB) and Support Vector Machines (SVM). These methods typically depend on simpler mathematical structures with fewer parameters^7–9^. Although they can perform adequately for basic classification tasks, they generally fall short in capturing complex patterns and subtle linguistic details present in natural language datasets, such as those encountered in inspection reports and defect records^10,11^.

In contrast, recent advancements in deep learning, specifically supervised learning methods based on Bidirectional Encoder Representations from Transformers (BERT), offer sophisticated architectures that incorporate multiple sequential layers. These layers introduce non-linear transformations during training, enabling BERT-based models to generate rich and contextually meaningful representations. Consequently, these models effectively address linguistic complexities, including diverse vocabularies, varied syntax structures, and stylistic variations commonly observed in construction-related textual data^12^.

However, numerous variations of BERT-based methods exist, and successfully deploying these models requires careful customization, extensive training, and systematic evaluation tailored specifically to the targeted classification task. These factors significantly impact key aspects of model performance, including accuracy and detection speed. Given these considerations, this study aims to develop and rigorously evaluate various BERT-based approaches for the automated classification of textual data, with a particular emphasis on detecting steel fire door defects.

The main contributions of this study are summarized as follows: (1) Real-world text data were collected from apartment complexes encompassing a total of 8,786 household units. (2) A robust classification framework was developed, identifying eight defect categories—seven common defects and one minor defect. (3) A comprehensive set of 1,458 predictive models were developed using five BERT-based methodologies, each extensively optimized through hyperparameter tuning. (4) 535 models based on other machine learning methods were optimized, and a detailed comparative analysis between these traditional machine learning models and the BERT-based models was conducted. (5) Based on resulting findings, the practical applications and implications for real-world construction management were thoroughly discussed.

## Literature review

### Typical process of BERT based text classification

The typical workflow for employing supervised deep learning models, particularly those based on the BERT family, involves several critical steps: target class definition, annotation, data cleaning, text vectorization, model training, and hyperparameter optimization^13^.

The process begins with clearly defining target classes from the raw textual data, a foundational step for effective model training^14^. For example, defects in fire doors may be broadly categorized into hardware faults and aesthetic faults, with potential for further subdivision into more detailed categories based on specific inspection requirements and intended use. After defining these target classes, an annotation step assigns precise labels to corresponding textual descriptions, creating structured datasets for model learning. Next, data cleaning is conducted to ensure textual data is consistent, relevant, and free from extraneous noise.

Following cleaning, text vectorization methods transform the textual data into numerical representations suitable for computational processing. BERT-based models play a crucial role here, producing context-aware vector representations that effectively capture complex linguistic patterns, significantly enhancing model performance^15^. Hyperparameter optimization systematically tunes model parameters such as learning rate, batch size, and the number of epochs to achieve optimal model performance. Each of these steps significantly influences overall model effectiveness^16^. This study specifically emphasizes evaluating the effectiveness of BERT-based vectorization methods for detecting defects in fire doors.

### Existing text classification methods

Various comparative studies have evaluated the performance of supervised learning methodologies within the construction industry, using both traditional machine learning and deep learning techniques. The methods employed in these studies, their achieved accuracies, and respective application contexts are summarized in Table 1.

Salama and El-Gohary^17^ compared machine learning methods, including SVM, NB, and Maximum Entropy (ME), for extracting rules from contractual texts, achieving the highest accuracy of 82% with ME. Goh and Ubeynarayana^18^ assessed methods such as SVM, Linear Regression (LR), and NB for construction accident classification, where SVM showed superior performance. Zhang et al.^19^ employed Decision Trees (DT), LR, and ensemble methods for classifying construction accident types, identifying Decision Trees as effective in their context.

Ul Hassan et al.^20^ developed supervised machine learning approaches to categorize textual descriptions into three project phases: design, construction, and operation and maintenance. Among the tested models—NB, SVM, Linear Regression (LR), K-Nearest Neighbor (KNN), DT, and Artificial Neural Networks (ANN)—LR performed best with an accuracy of 94.12%. Luo et al.^21^ also evaluated traditional models such as SVM, NB, and LR alongside Convolutional Neural Networks (CNN) for accident type classification, with CNN delivering the highest accuracy of 76%. Wang et al.^22^ proposed both machine learning and deep learning approaches, including DT, Random Forest (RF), NB, SVM, and CNN with Attention (CNN-AT), to categorize construction defects from daily supervisory reports. Among these, NB demonstrated notable performance with an accuracy of 98%.

Moreover, recent studies highlighted transformer-based methods’ superior capability in text classification tasks. Fang et al.^23^ employed deep learning models, including FastText, TextCNN, TextCNN combined with Bidirectional Gated Recurrent Units (BiGRU), Text Recurrent Convolutional Neural Networks (TextRCNN), and BERT, to classify near-miss incidents, with BERT obtaining the highest accuracy of 86.91%. Yang et al.^24^ applied deep learning methods such as CNN and Generative Pre-trained Transformer 2 (GPT-2) to classify facility defects, achieving the highest accuracy of 90.72% using a CNN-based model. Additionally, Wang et al.^25^ evaluated transformer architectures such as BERT-Bidirectional Long Short-Term Memory (BiLSTM) and BERT-LSTM-CRF for explicit safety knowledge extraction from regulatory texts, obtaining the highest accuracy of 91.74% with the BERT-BiLSTM-CRF method.

Tian et al.^26^ compared transformer-based approaches such as BERT-Graph Convolutional Network (GCN) and BiLSTM for safety hazard classification, achieving accuracy up to 86.56%. Jianan et al.^27^ evaluated transformer architectures, including RoBERTa and Longformer-RoBERTa, for classifying knowledge types from construction consulting standards, with the Longformer-Robustly Optimized BERT Approach (RoBERTa) variant reaching the highest accuracy of 91.65%. In addition, a systematic assessment by Zhong et al.^28^ contrasted classical classifiers (SVM, NB, LR, DT, KNN) with neural architectures (TextCNN, TextCNN–LSTM, RCNN, Transformer) for construction-dispute classification; TextCNN achieved the best result at 65.09% accuracy.

These findings indicate that no single algorithm consistently outperforms others across all scenarios, underscoring that the optimal model choice heavily depends on the specific classification task and its context. Currently, no research has been conducted on automated monitoring of fire door defects in buildings. Despite the proven effectiveness of transformer-based methods, particularly BERT variants, their application to classifying fire door defects from inspection reports remains underexplored. To address this research gap, this study proposes and evaluates multiple transformer-based models (BERT, RoBERTa, A Lite BERT (ALBERT), Distilled BERT (DistilBERT), and XLNet). Additionally, traditional machine learning algorithms (ANN, SVM, DT, RF, LR) and other deep learning approaches (1D CNN and LSTM) are comprehensively assessed and compared.

**Table 1:** Summary of reviewed studies.

Authors	Applications	Traditional method	Deep learning
Salama and El-Gohary^17^	Rule extractions	SVM: 70 NB: 44 **ME: 82**	-
Goh and Ubeynarayana^18^	Construction accident types	**SVM: 67** LR: 35 RF: 42 KNN: 44 DT: 53 NB: 41	-
Zhang et al.^19^	Construction accident types	DT: 52 KNN: 53 NB: 44 SVM: 58 LR: 50 Ensemble: 52 **Optimized ensemble: 68**	-
Ul Hassan et al.^20^	Design-build contract requirements	SVM: 93.17 NB: 87.48 **LR: 94.18** DT: 89.38 k-NN: 92.03 ANN: 92.80	-
Luo et al.^21^	Construction accident types	SVM: 73 NB: 54 LR 66	**CNN: 76**
Wang et al.^22^	Construction defects categories	DT: 92% RF: 91% **NB: 98%** SVM: 85%	CNN-AT: 92%
Fang et al.^23^	Safety requirements on construction sites	-	TextCNN with BiGRU: 75.31 TextCNN: 74.82 BiGRU with Attention: 75.18 TextRCNN: 75.26 **BERT: 86.91**
Yang et al.^24^	Facility defects in residential buildings	-	CNN (FastText): 87.44 CNN (Word2Vec): 88.25 BERT: 89.62 ELECTRA: 89.47 GPT2: 89.68 **CNN (FastText and Word2Vec): 90.72**
Wang et al.^25^	Safety knowledge mapping		BERT-BiLSTM: 75.48 BERT-LSTM-CRF: 91.08 **BERT-BiLSTM-CRF: 91.74**
Tian et al.^26^	Safety hazard classification		TextCNN: 79.56 BiLSTM: 80.42 **BERT-GCN- BiLSTM: 86.56** BERT-GCN: 82.34
Jianan et al.^27^	Knowledge types of consulting standards		BERT: 90.17 RoBERTa: 90.94 **Longformer-RoBERT: 91.65** Longformer-RoBERT with CNN: 86.7 Longformer-RoBERT with LSTM: 83.81
^28^	Construction contract disputes	SVM: 62.65 NB: 50.55 LR: 62.50 DT: 57.90 k-NN: 44.85	**TextCNN: 65.99** TextCNN + LSTM: 63.20 R-CNN: 57.91 Transformer: 54.80
Aim of this research	Fire door defect classification	ANN, SVM, DT, LR, 1D CNN, and LSTM	BERT, RoBERTa, ALBERT, DistilBERT, and XLNet

## Proposed approach

**Fig. 1:**
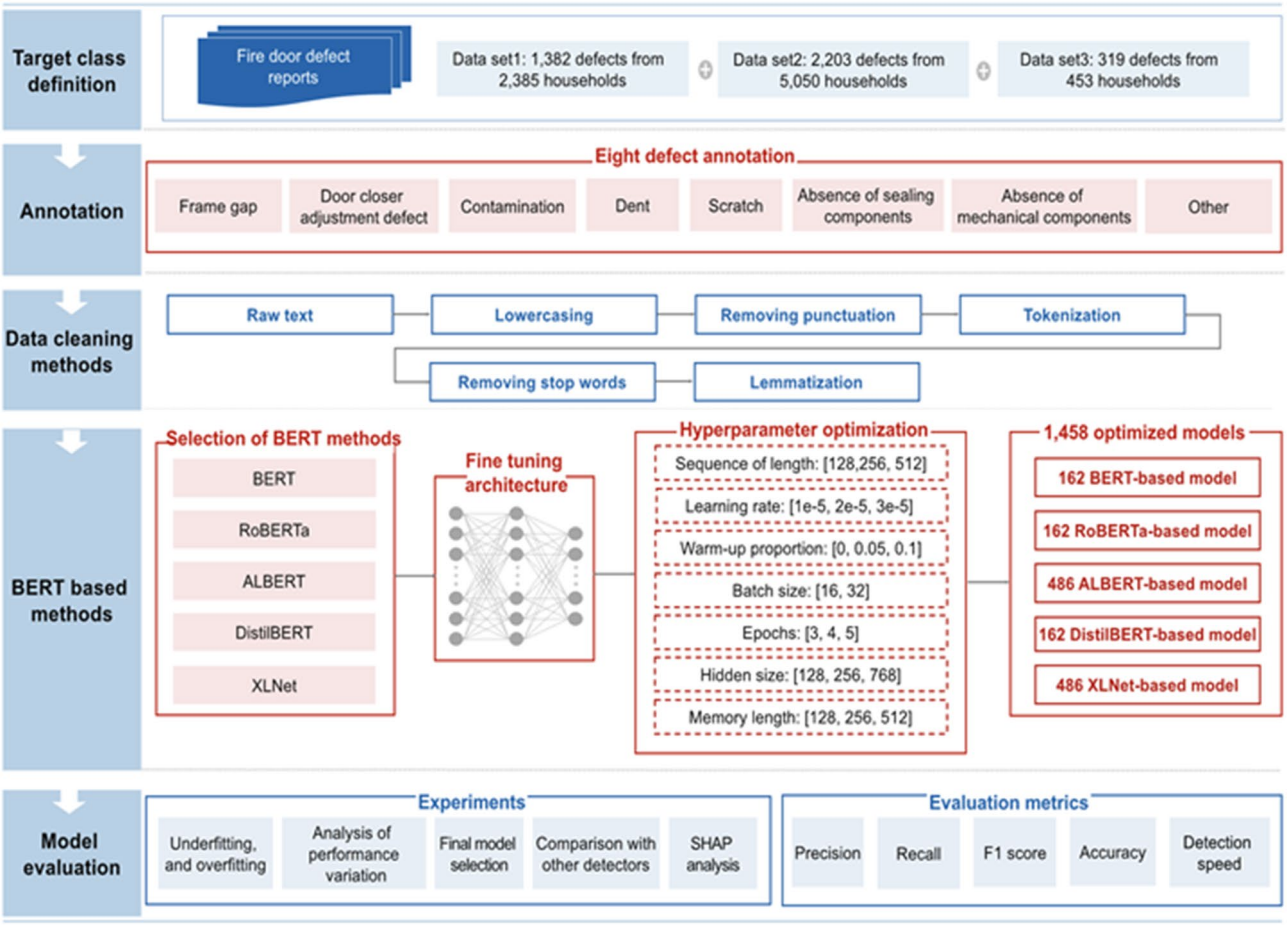
Development workflow of the proposed method.

As illustrated in Fig. 1, the proposed method consists of the following steps: First, reports describing fire door defects are collected from real households and categorized based on various defect types, such as frame gap and contamination. Next, each instance within the collected dataset is systematically annotated according to the corresponding defect categories. The dataset is then preprocessed through sequential cleaning steps, including lowercasing and lemmatization. Subsequently, five different transformer-based models (BERT, RoBERTa, ALBERT, DistilBERT, XLNet) are developed by fine-tuning their architectures and systematically optimizing hyperparameters, resulting in a total of 1,458 generated models. These transformer-based models are carefully evaluated to identify underfitting and overfitting issues, and their performance is then compared against other established text classification methods, comprising an additional 535 models. Multiple evaluation metrics are utilized for a comprehensive comparative analysis. Each of these steps is thoroughly detailed in the subsequent sections.

### Target class definition

In this research, excluding the category labeled ‘others’, seven distinct defect types were determined based on fire door inspection records. These include gaps in the frame, defects related to door closer adjustments, contamination issues, dents, scratches, missing sealing elements, and missing mechanical operation components.

**Fig. 2:**
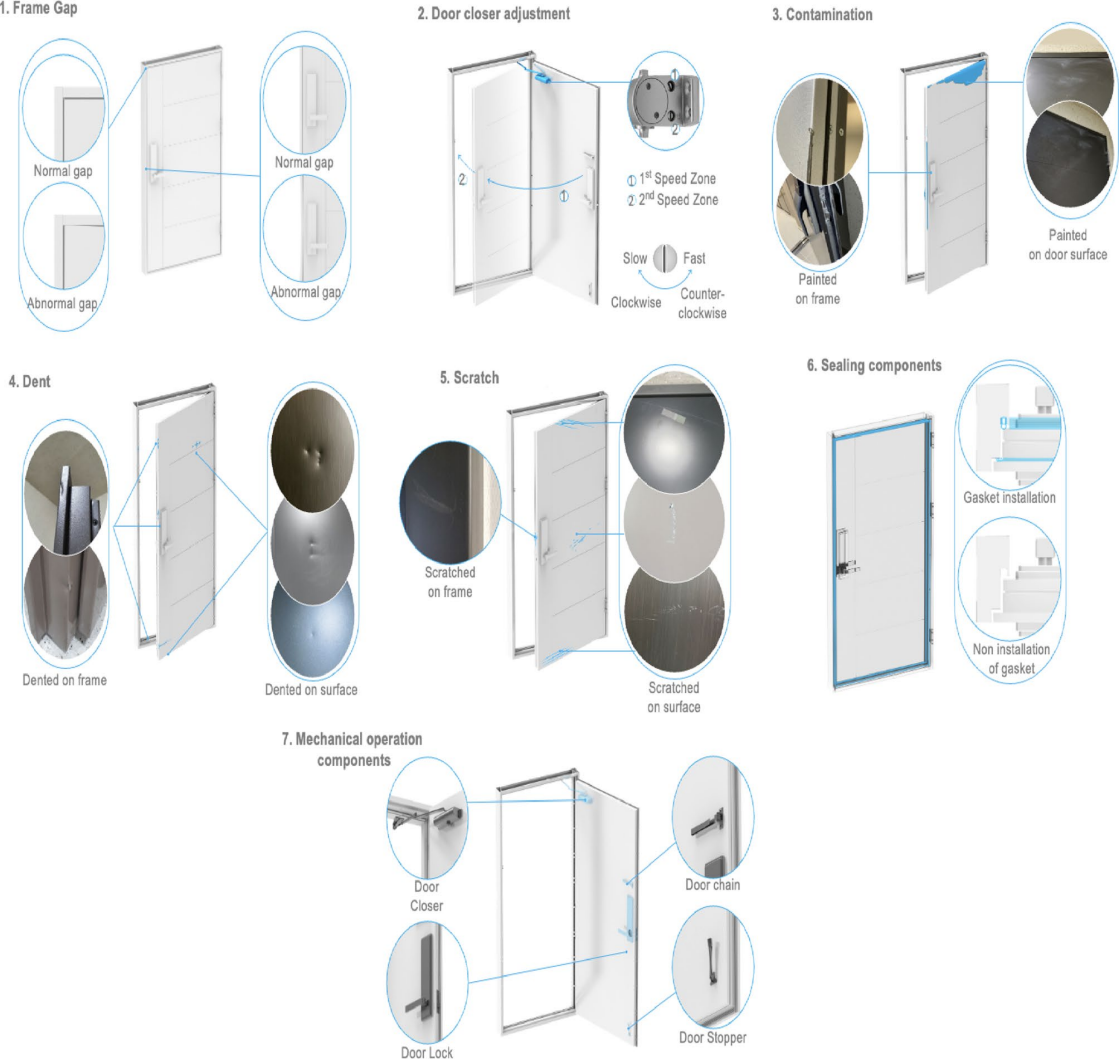
Visual examples of seven defects in fire door.

Each defect type is visually demonstrated in Fig. 2. Frame gap defects occur as horizontal or vertical separations between the door and its frame. Defects in door closer adjustments involve incorrect calibration of the closer’s two distinct speed zones, each controlled via an adjustment screw. Rotating this screw clockwise decreases the door’s closing speed, whereas rotating it counter-clockwise accelerates the closing. Therefore, precise adjustment is essential for effective operation.

Contamination, dents, and scratches typically affect door or frame surfaces. Deficiencies classified under missing sealing elements usually pertain to the absence of critical gaskets required for containing smoke and fire effectively. Lastly, defects classified as missing mechanical operation components include absent essential hardware such as door closers, digital locks, hinges, or door stoppers.

### Annotation

**Fig. 3:**
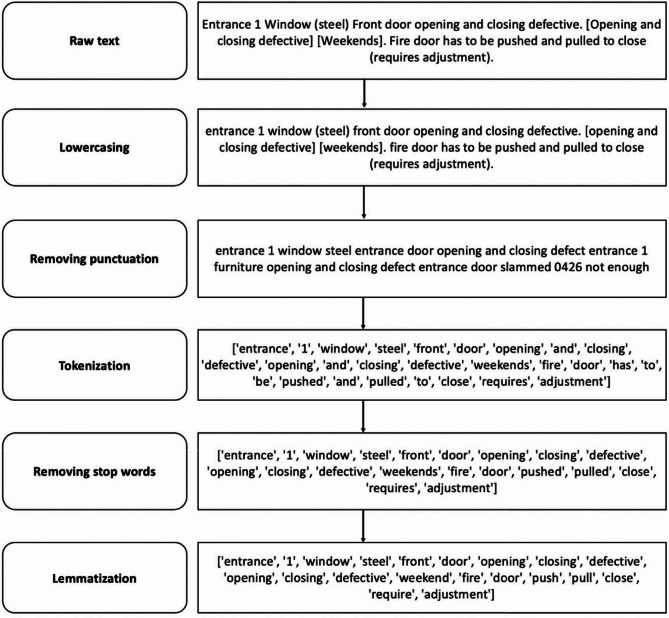
Data cleaning methods with examples.

After visually inspecting fire doors for defects, inspectors first document their findings manually in handwritten reports. Sample statements extracted from these handwritten records are presented in Table 2. Each fire door can have several distinct defects, and each defect is individually recorded. Subsequently, the handwritten observations are converted into digital form, as shown earlier in Fig. 3. Finally, these digital sentences are annotated with appropriate labels that identify the specific categories of defects.

**Table 2:** Labeling basis on seven types of classification.

Category	Description	Example sentences
Frame gap	A situation where an uneven gap exists between the door and its frame	Balcony 3 window (steel), outdoor unit room fire door not fixed properly 23.12.14 Balcony 2 wall not fixed properly, outdoor unit room door shaking
Door closer adjustment	Issues arising from incorrect calibration or adjustment of the door closer mechanism	Entrance 1 window (steel) door closer construction defective Entrance 1 window construction defective Front door door closer tension adjustment/0504 insufficient/adjusted
Contamination	Presence of foreign substances such as paint, oil, or other contaminants on the door surface	Entrance 1 window (steel) lower seal damaged Entrance 1 door frame contaminated/0429 completion cleaning -> window steel transfer
Dent	Physical impairment characterized by indentations or depressions in the door’s surface	Entrance 1 window (steel) lower seal damaged [Lower seal] Lower seal dented
Scratch	Superficial damage presenting as lines or marks on the surface	Entrance 1 window (steel) Damage to the front door 23.12.18 [Painting] Damage to the entrance 1 window Scratch damage to the front door Unprocessed in June Request for quick processing
Sealing components	Missing sealing elements, primarily gasket materials, essential for effective sealing	Balcony 3 window (steel) gasket not installed. 24.1.24 Balcony 2 window not installed. Rubber packing under the protective wall is not installed, so it does not block wind noise and cannot be sealed. Request for quick construction.
Mechanical operation components	Missing or absent hardware components directly influencing door functionality, including door closers, digital locks, hinges, and stoppers	Entrance 1 Window (steel) Entrance door not constructed Exterior fire door stopper not installed Floor behind the door
Others	Any defects not categorized in the above seven classifications are grouped as “others.”	Entrance 1 window (steel), door closer construction defective [Door Closer] 23.08.21 Entrance 1 window construction defect, oil continues to build and drip. I tried calling, but no response.

### Data cleaning methods

In this study, the five data preprocessing methods were employed to refine textual information. The impact of these preprocessing techniques on raw text data transformation is demonstrated with an example in Fig. 3:

1. Lowercasing: Converts all text characters to lowercase. This standardizes words such as “Fire door” and “fire door”, eliminating unnecessary distinctions and simplifying the dataset.
2. Removing punctuation: Exclude punctuation marks (e.g., commas, question marks, periods) from the text. For many classification tasks, punctuation adds little semantic value; eliminating it reduces noise and yields a cleaner, more consistent input.
3. Tokenization: Breaks down text into smaller components known as tokens, typically words. Tokenization converts raw text into structured units that can then be represented numerically, making them suitable for processing by algorithms used in text classification.
4. Removing stop words: Excludes frequently occurring words such as “and,” “the,” “is,” and “to,” which usually provide minimal informational value. Removing these stop words considerably reduces vocabulary size and data complexity, facilitating more efficient processing.
5. Lemmatization: Reduces words to their fundamental or dictionary forms, known as lemmas, based on grammatical usage and context. For example, the words “pushed” and “pulled” would become “push” and “pull,” respectively. Unlike basic truncation, lemmatization identifies accurate root forms, ensuring consistent data representation.

### BERT-based methods

**Fig. 4:**
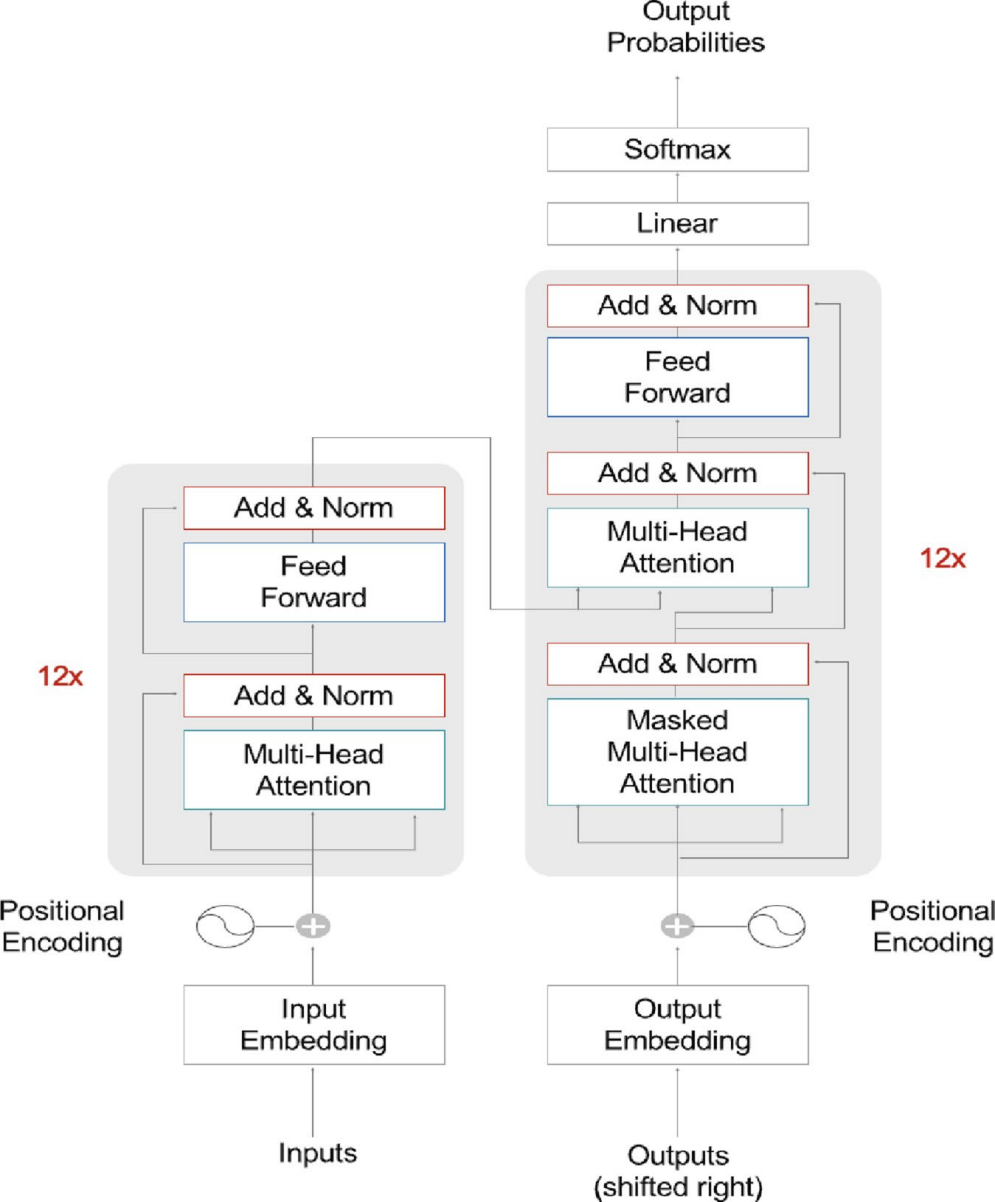
Workflow of transformer architecture.

The transformer architecture, proposed by Vaswani et al.^29^, employs stacked self-attention layers and fully connected layers organized into encoder and decoder modules. Unlike traditional sequence models, transformers leverage self-attention mechanisms instead of recurrent or convolutional operations, enabling the efficient modeling of long-range dependencies within textual data^30,31^. The overall transformer architecture, which consists of multiple layers of self-attention and feed-forward neural networks in both encoder and decoder blocks, is illustrated in Fig. 4.

To incorporate positional information, positional encodings are combined directly with input embeddings at the base of both encoder and decoder modules. These positional encodings match the dimensionality ($\:{d}_{model}$) of the embeddings, allowing seamless integration. Positional encodings use sinusoidal functions at different frequencies, as defined by Eqs. (1) and (2):

1 $$\:PE\left(pos,\:2i\right)=\:\text{s}\text{i}\text{n}\left(\frac{pos}{{\text{10,000}}^{2i/{d}_{model}}}\right)$$

2 $$\:PE\left(pos,\:2i+1\right)=\:\text{c}\text{o}\text{s}\left(\frac{pos}{{\text{10,000}}^{2i/{d}_{model}}}\right)$$

Here, *pos* represents the position in the sequence, and *i* indicates the embedding dimension. Each dimension generates a distinct sinusoidal waveform. The Transformer encoder and decoder each consist of identical layers. Each encoder layer includes a multi-head self-attention mechanism followed by a fully connected feed-forward network, while each decoder layer contains these components plus an additional multi-head attention layer focused on encoder outputs^32^.

Scaled Dot-Product Attention (Fig. 6) processes queries (Q), keys (K), and values (V), with attention weights calculated by scaling dot products between queries and keys by the square root of the key dimension ($\:{d}_{k}$), then applying a softmax function, as expressed in Eq. (3):

3 $$\:Attention\left(Q,\:K,\:V\right)=\:\text{s}\text{o}\text{f}\text{t}\text{m}\text{a}\text{x}\left(\frac{{QK}^{T}}{\sqrt{{d}_{k}}}\right)V$$

**Fig. 5:**
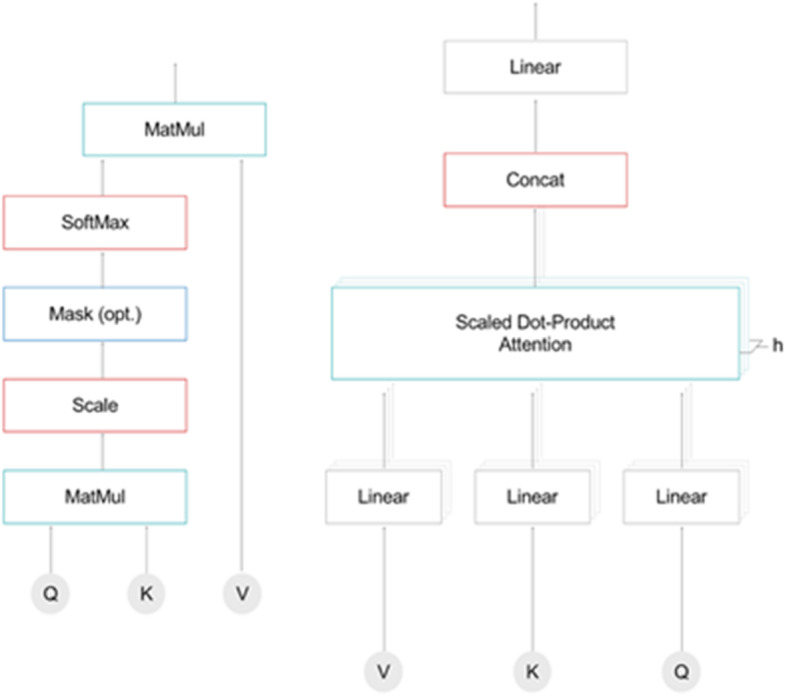
Scaled dot and multi-head attention.

In multi-head attention, instead of utilizing a single attention operation with *d*_model-dimensional queries, keys, and values, multiple parallel attention heads (h) are employed as shown Fig. 5, each applying unique linear projections to produce queries, keys, and values of dimensions $\:{d}_{q}$, $\:{d}_{k}$, and $\:{d}_{v}$, respectively^33^. Multi-head attention enhances performance by parallelizing attention computations across multiple attention heads, each using unique linear projections of queries, keys, and values^34^.

Building upon the transformer architecture, numerous variants have emerged, each tailored to specific tasks and use cases. These variants primarily differ in their pre-training strategies, parameter efficiency, and internal architectural enhancements. The following subsections provide detailed discussions of prominent BERT variants, with their key distinctions clearly summarized in Table 3.

**Table 3:** Summary of distinct characteristics in each method.

Aspect	BERT	RoBERTa	ALBERT	DistilBERT	XLNet
Training Objectives	Masked Language	Masked Language	Masked Language	Masked Language	Permutation Language
Pre-training Method	Autoencoding	Autoencoding	Autoencoding	Distillation	Autoregressive
Autoregressive	No	No	No	No	Yes
Factorized Embedding	No	No	Yes	No	No
Computational Resources	More demanding	More demanding	Less demanding	Less demanding	More demanding

#### BERT

BERT is an encoder-only Transformer trained with Masked-Language Modeling (MLM) in an autoencoding regime^35^. A small subset of tokens is replaced by a mask token, and the network recovers the originals using bidirectional self-attention, which integrates left and right context within each encoder block. The model employs subword tokenization (e.g., WordPiece), learned token and positional embeddings, and a stack of multi-head self-attention and position-wise feed-forward layers with residual connections and layer normalization. For sequence classification, a task-specific affine transformation with softmax is applied to the sequence-level [CLS] representation (classification token), and all parameters are fine-tuned jointly. Due to its parameter count and the quadratic cost of self-attention in sequence length, BERT is generally more demanding computationally than lighter variants.

#### RoBERTa

RoBERTa preserves BERT’s MLM objective and autoencoding pre-training while emphasizing optimization and scale through dynamic masking, larger mini-batches, longer training schedules, and larger corpora, alongside implementation refinements that stabilize learning^36^. Downstream use follows the same encoder-only architecture and fine-tuning protocol as BERT; a task-specific affine transformation with softmax is applied to the [CLS] representation, and all parameters are fine-tuned jointly. Given its reliance on aggressive scaling, RoBERTa typically matches or exceeds BERT’s accuracy at similar model sizes while being equally or more demanding to pre-train.

#### ALBERT

ALBERT targets parameter efficiency within the same MLM/autoencoding paradigm by introducing factorized embedding parameterization, which decouples the vocabulary embedding dimension from the hidden size used in Transformer layers, and by applying cross-layer parameter sharing to reduce the number of unique weights^37^. These design choices substantially lower memory usage and improve throughput with limited accuracy loss. Fine-tuning mirrors BERT; a task-specific affine transformation with softmax is applied to the [CLS] representation, and all parameters are fine-tuned jointly. With far fewer parameters, ALBERT is less demanding in both memory and inference latency, making it suitable for resource-constrained settings.

#### DistilBERT

DistilBERT compresses BERT via knowledge distillation while retaining the MLM/autoencoding objective. A smaller student model with fewer encoder layers is trained to match a larger teacher’s softened outputs (and, in some implementations, intermediate representations), typically combining a distillation term with the MLM loss^38^. Architectural simplifications further reduce complexity without altering the encoder-only design. In downstream tasks, a task-specific affine transformation with softmax is applied to the [CLS] representation, and all parameters are fine-tuned jointly. This compression yields markedly lower latency and memory usage with modest accuracy trade-offs, rendering DistilBERT less demanding than full-size BERT at inference.

#### XLNet

XLNet replaces MLM with Permutation Language Modeling (PLM)—an autoregressive objective that maximizes the expected likelihood over all factorization orders of a sequence^39^. Built on Transformer-XL, it incorporates segment-level recurrence and relative positional encodings to capture long contexts efficiently, and employs two-stream attention to condition on permuted “future” tokens without information leakage. For sequence classification, implementations use a sequence-level summary token (analogous to [CLS]); a task-specific affine transformation with softmax is applied to this sequence-level representation, and all parameters are fine-tuned jointly. Owing to the PLM objective and long-context machinery, XLNet typically incurs a higher computational burden, comparable to or above BERT/RoBERTa, while providing strong language-understanding performance.

#### Fine tuning of architectures

To utilize BERT-based models for classification tasks, a FC (Fully Connected) classification layer is typically added on top of the transformer architecture. The embeddings generated by the BERT model are passed into an FC classification head, which generally consists of one dense hidden layer with 128 units, followed by a dropout layer with a dropout rate of 0.3. Finally, a dense output layer with softmax activation classifies these embeddings into distinct categories, ensuring reliable accuracy and efficient computational performance^40–42^. In this research, the classifier head is specifically configured to classify fire-door conditions into eight distinct categories, as described in Table 2.

#### Optimization of hyperparameters

Selecting optimal hyperparameters is essential for achieving high model performance; however, exhaustive hyperparameter optimization can be computationally expensive^43–45^. A practical strategy to address this is to define hyperparameter ranges based on empirical results and previous studies^46–48^. Accordingly, hyperparameter ranges and specific values were established, resulting in the generation of 1,458 model configurations. Detailed information regarding the selected hyperparameters for each method and the corresponding number of generated models is clearly summarized in Table 4.

**Table 4:** Detail of used hyperparameters in each method.

Algorithm	Common hyperparameters	Specific hyperparameters	Numbers of models	Best hyperparameter
BERT	Sequence length [128, 256, 512], Learning rate: [1e-5, 2e-5, 3e-5], Warm-up proportion: [0, 0.05, 0.1], Batch size: [16, 32] Epochs: [3, 4, 5],	-	162	Sequence length: 512, Learning rate: 2e-5, Warm-up proportion: 0.05, Batch size: 32, Epochs: 4
RoBERTa		-	162	Sequence length: 512, Learning rate: 1e-5, Warm-up proportion: 0.1, Batch size: 32, Epochs: 5
ALBERT		Hidden size: [128, 256, 512]	486	Sequence length: 512, Learning rate: 2e-5, Warm-up proportion: 0.05, Batch size: 16, Epochs: 5, Hidden size: 512
DistilBERT		-	162	Sequence length: 256, Learning rate: 5e-5, Warm-up proportion: 0.05, Batch size: 32, Epochs: 4
XLNet		Memory length: [128, 256, 512]	486	Sequence length: 256, Learning rate: 3e-5, Warm-up proportion: 0, Batch size: 16, Epochs: 5, Memory length: 256

### Model evaluation

#### F1 score and accuracy

In classification tasks, both the F1 score and accuracy are widely used metrics to evaluate model correctness. The F1 score integrates precision and recall into a single metric, balancing the model’s ability to correctly detect relevant cases (recall) and its effectiveness in avoiding false alarms (precision). A detailed explanation of the F1 score is provided by^49,50^. A high F1 score indicates that the model effectively identifies faults while minimizing false positives and false negatives, crucial for reliable system operations. Accuracy measures the proportion of correctly classified instances over the total number of instances, providing a straightforward assessment of overall model correctness^51,52^. Both metrics are important for evaluating model performance, especially in scenarios where misclassification can lead to significant operational consequences.

#### Detection speed

The detection speed refers to the computational time a model takes to process a single input instance^53^. In this study, the detection speed is reported in terms of instances processed per second, reflecting the efficiency of each transformer-based method when analyzing textual descriptions of fire door defects.

## Experiment

### Dataset preparation

#### Raw data collection

The dataset analyzed in this research regarding fire door defects was gathered from three residential apartment complexes in South Korea, each constructed by a different developer. All complexes utilized standardized steel fire-rated doors. Inspectors systematically checked a total of 8,786 households, recording identified defects in fire doors. Through these inspections, 4,212 separate defects were documented. Further details about the households inspected and the distribution of defects among the three companies are summarized in Table 5. Initially, inspection personnel manually documented the defects in Korean, subsequently converting these handwritten reports into digital form. For the purpose of this study, the original Korean descriptions were translated into English for clear presentation.

**Table 5:** Characteristics of defect datasets recorded by companies.

Construction companies	Number of households	Number of defects	Work experience of inspectors	Household Dissatisfaction (Yes/No)
J	2,385	1,322	More than 3 years	Yes
B	5,050	2,203	Less than 6 months	No
Y	453	319	Less than 1 year	No
C	925	368	Less than 6 months	No

**Fig. 6:**
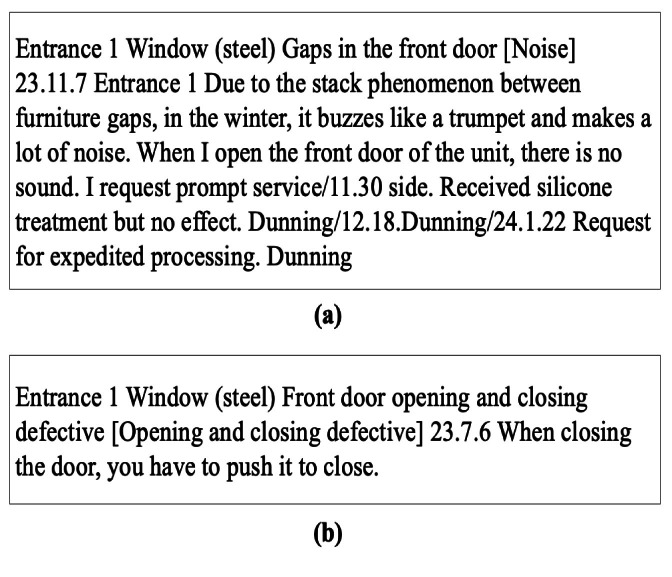
Examples of defect descriptions by work experience of inspectors.

The degree of specificity in defect descriptions differed significantly across various construction firms, resulting in diverse classification methods. Several key factors influencing textual differences among companies were evident from the collected data. A primary factor impacting these variations was the training level of the inspectors; individuals with more specialized training often described defects using technical vocabulary. Experienced inspectors, in particular, utilized precise and technically advanced terminology. Figure 6 demonstrates this contrast by comparing descriptions from Company A and Company B. Specifically, in Fig. 6(a), a defect is articulated explicitly using the technical term “stack phenomenon,” reflecting a detailed, technical perspective. Conversely, Fig. 6(b) illustrates the same defect described by Company B using simpler, less technical wording.

**Fig. 7:**
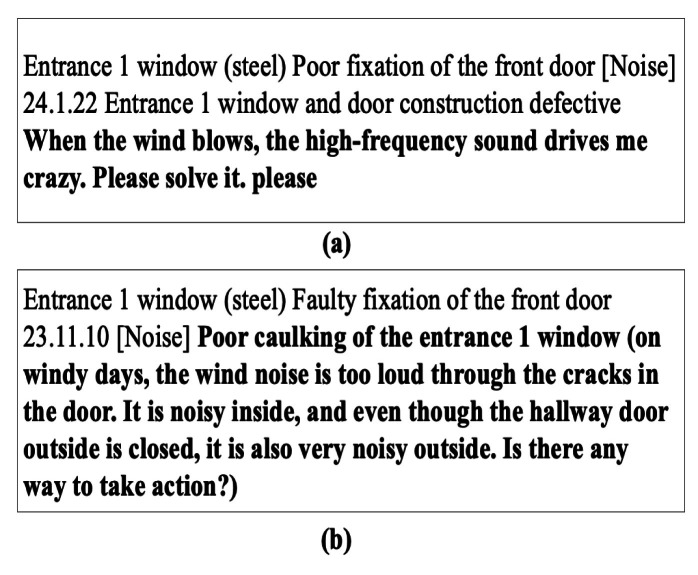
Example of variability of language.

Furthermore, in Apartment A, residents’ feedback regarding dissatisfaction was collected via a mobile app and subsequently integrated with defect descriptions to improve the overall accuracy and comprehensiveness of defect assessments. An illustrative example of this combined approach appears in Fig. 7, where the residents’ dissatisfaction is emphasized in bold text for clarity. In addition to inspector training and resident input, other elements influencing the detail level of defect descriptions included the duration of inspections, availability of inspection staff, and budgetary limitations.

**Fig. 8:**
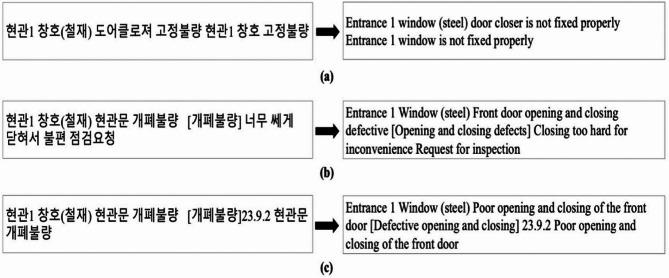
Example of description including dissatisfaction by household.

Due to the inherent flexibility and variability of linguistic expressions in Korean, translating textual descriptions into English required careful attention to maintain the original nuances. Figure 8 illustrates this challenge by showcasing varied expressions related to defects in window and door operation mechanisms. Therefore, the distinct variations originally present in the Korean descriptions were intentionally retained in the English translations to authentically represent this linguistic diversity.

#### Annotation

In this study, annotation of the raw textual data was carried out according to criteria specified in Table 3, with the purpose of classifying defects explicitly associated with fire doors. Three annotators, each with over a decade of experience dealing with fire door defects and extensive expertise in construction management, manually labeled the data by recording defect details into an Excel spreadsheet. The completed labeled dataset can be obtained by contacting the corresponding author.

#### Data split

Following annotation, the entire dataset, consisting of 4,212 instances, was randomly divided into three distinct subsets: a training set comprising 2,527 instances (approximately 60% of the data) for model development, a validation set containing 842 instances (approximately 20% of the data) for selecting the optimal model, and a test set with 843 instances (approximately 20% of the data) for evaluating the performance of the final model on unseen data. To address potential biases or underrepresentation of smaller classes, such as contamination, stratified sampling was applied. Stratified sampling ensures that each subset maintains class proportions consistent with those found in the overall dataset^54^. For example, if a particular class represents 5% of the complete dataset, it will similarly constitute about 5% within each subset. Detailed distributions for each subset are provided in Table 6.

**Table 6:** Detailed distribution of data sets.

Types of defects	Number of data			
	Training	Validation	Test	Total
Frame gap	605	201	202	1,008
Door closer adjustment	328	109	109	546
Contamination	57	19	19	95
Dent	155	52	51	258
Scratch	152	51	51	254
Sealing components	251	83	84	418
Mechanical operation components	863	288	288	1,439
Others	116	39	39	194
Total	2,527	842	843	4,212

### Experimental settings

All experiments were conducted on a system operating on Windows 10, equipped with an Intel Core i7-7700HQ CPU (2.80 GHz, 8 threads), an NVIDIA GeForce GTX 3080 Ti GPU, and sufficient memory to handle computational tasks effectively. The implementations utilized the TensorFlow framework for developing and executing the deep learning models.

## Results and discussion

In this research, the performance of classification models was evaluated across eight defect categories. For clarity, these defect categories are defined as follows: Class1 – Frame gap, Class2 – Door closer adjustment, Class3 – Contamination, Class4 – Dent, Class5 – Scratch, Class6 – Sealing components, Class7 – Mechanical operation components, and Class8 – Others.

### Underfitting, and overfitting

In this study, the training and validation loss curves were analyzed to identify potential issues related to underfitting or overfitting during model training. Multiple hyperparameter combinations involving different batch sizes and epochs were explored, affecting the total number of training iterations. The scenario with the maximum number of iterations (batch size of 16, epochs 4, and a dataset of 2,527 training instances, resulting in 4,992 iterations) was specifically chosen, as it provided the richest insights into model convergence behavior compared to scenarios with fewer iterations (e.g., batch size of 32, epochs 4, with only 624 iterations).

**Fig. 9:**
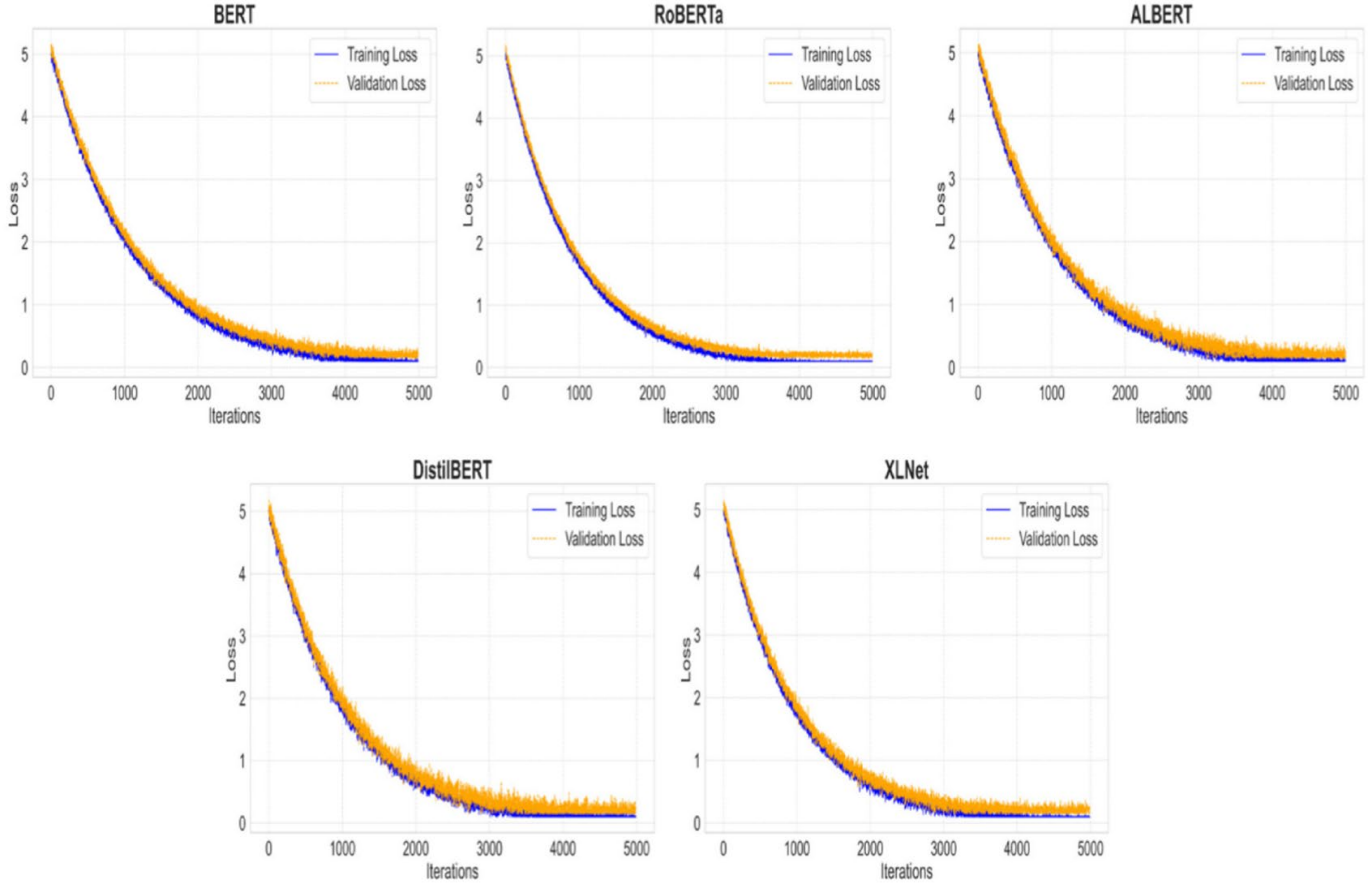
Training and validation loss curves for each transformer-based method over 5 epochs (4,992 iterations).

Figure 9 illustrates the training and validation loss curves for each model under this maximum iteration scenario. All models exhibited a clear and consistent decrease in both training and validation losses as training progressed, indicating effective and steady learning across the iterations. The consistent decline in training loss confirms that none of the models experienced significant underfitting. Furthermore, the close alignment and concurrent downward trends observed in both training and validation loss curves suggest that overfitting was minimal or non- existent.

### Analysis of performance variation by methods

**Fig. 10:**
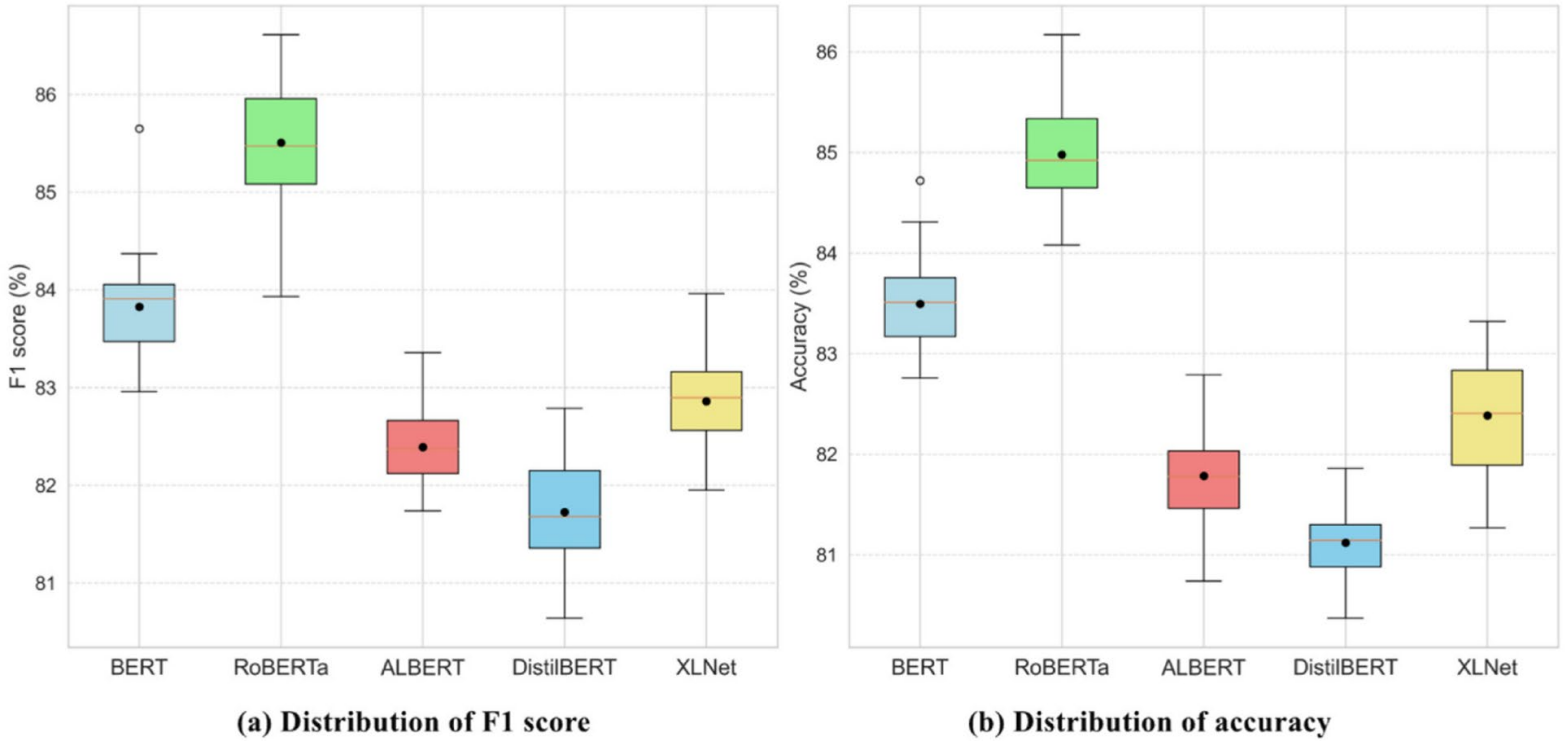
Boxplot of F1 score, and accuracy across methods by each hyperparameter.

Figure 10 presents boxplots illustrating the distribution of F1-score, and accuracy metrics for five transformer-based models by hyperparameter combinations. A preliminary visual analysis shows that RoBERTa consistently achieves the highest accuracy and F1-score, with its boxplots positioned distinctly higher and demonstrating narrower interquartile ranges, indicating strong and stable performance. In contrast, DistilBERT exhibits lower overall performance, as indicated by boxplots located at lower positions, reflecting weaker predictive capability. ALBERT and XLNet demonstrate moderate performance, with tightly grouped distributions suggesting stable yet moderate performance across parameter combinations. BERT shows intermediate performance but has slightly wider distribution ranges, indicating variability in response to hyperparameters.

Detailed statistical analysis as shown in Table 7 further substantiates these observations. RoBERTa achieves the highest mean accuracy (84.98%) and F1-score (85.51%), confirming superior and consistent performance. DistilBERT displays the lowest mean accuracy (81.12%) and F1-score (81.73%), reinforcing its lower overall performance despite minimal variance (std = 0.37). ALBERT and XLNet yield moderate results, with stable but lower averages, highlighting less sensitivity to hyperparameter variations. BERT exhibits moderate performance but higher variability, suggesting sensitivity to specific hyperparameters.

**Table 7:** Statistics of F1 score and accuracy.

Model	Min	25%	Median	Mean	75%	Max	Std
F1 score							
BERT	82.96	83.47	83.91	83.83	84.06	85.65	0.52
RoBERTa	83.93	85.08	85.47	85.51	85.96	86.61	0.59
ALBERT	81.74	82.12	82.38	82.39	82.66	83.36	0.39
DistilBERT	80.64	81.36	81.68	81.73	82.15	82.79	0.57
XLNet	81.95	82.56	82.9	82.86	83.16	83.96	0.43
Accuracy							
BERT	82.76	83.17	83.51	83.49	83.76	84.72	0.46
RoBERTa	84.08	84.65	84.92	84.98	85.34	86.17	0.48
ALBERT	80.74	81.46	81.78	81.79	82.04	82.79	0.41
DistilBERT	80.37	80.88	81.15	81.12	81.3	81.86	0.37
XLNet	81.27	81.89	82.4	82.39	82.84	83.32	0.57

As shown in Table 8, per-class performance is reported for the best model under each method. RoBERTa shows the strongest overall results, with the highest mean accuracy (85.01%) and mean F1 score (85.47%) among the evaluated models. Specifically, RoBERTa achieved superior precision (up to 99.78%) and recall (up to 99.13%) in certain classes, highlighting its strong predictive capability and consistent reliability. BERT exhibited moderate performance, with an average accuracy of 83.36% and mean F1-score of 83.83%, although it notably struggled with Class 8 (F1-score: 65.87%). ALBERT had stable but lower average performance (accuracy: 81.79%, F1-score: 82.31%), suggesting reliability but somewhat limited predictive accuracy.

DistilBERT showed the lowest performance across all models, with a mean accuracy of 81.12% and mean F1-score of 81.70%, indicating limited effectiveness compared to other methods. XLNet exhibited moderate performance (accuracy: 82.29%, F1-score: 82.86%), placing it between ALBERT and BERT in overall capability.

**Table 8:** Best model in each method on validation data.

Category	Class1	Class2	Class3	Class4	Class5	Class6	Class7	Class8	Average
BERT									
Precision	90.45	86.12	76.05	80.91	79.62	95.87	98.45	65.87	84.17
Recall	89.52	85.36	75.23	79.84	78.52	95.26	97.87	64.59	83.27
F1 score	89.98	85.74	75.64	80.37	79.07	95.56	98.16	65.22	83.72
Accuracy	90.23	85.93	75.03	79.7	78.81	95.13	97.73	64.32	83.36
RoBERTa									
Precision	92.87	88.58	78.92	83.71	82.15	98.25	99.78	69.23	86.69
Recall	91.22	86.17	75.92	81.15	79.61	96.55	98.75	66.14	84.44
F1 score	92.04	87.36	77.39	82.41	80.86	97.39	99.26	67.65	85.55
Accuracy	91.52	87.02	76.84	81.54	80.21	97.12	99.13	66.72	85.01
ALBERT									
Precision	89.13	84.75	74.8	79.85	78.1	94.92	97.25	64.15	82.87
Recall	88.21	83.26	73.57	78.28	76.66	94.51	96.59	62.98	81.76
F1 score	88.67	84.00	74.18	79.06	77.37	94.71	96.92	63.56	82.31
Accuracy	88.34	83.62	73.32	78.13	77.01	94.22	96.41	62.73	81.72
DistilBERT									
Precision	88.73	84.28	74.25	78.95	77.35	94.62	96.83	63.58	82.32
Recall	87.52	82.86	72.95	77.32	75.84	93.85	96.06	62.25	81.08
F1 score	88.12	83.56	73.59	78.13	76.59	94.23	96.44	62.91	81.70
Accuracy	87.72	83.03	72.93	77.22	76.21	93.72	95.93	61.92	81.09
XLNet									
Precision	89.93	85.44	75.37	80.12	78.88	95.37	97.89	65.22	83.53
Recall	88.83	84.53	74.27	78.81	77.4	94.12	97.57	63.99	82.44
F1 score	89.38	84.98	74.82	79.46	78.13	94.74	97.73	64.60	82.98
Accuracy	88.92	84.72	73.91	78.62	77.71	94.02	96.81	63.62	82.29

### Final model selection

As shown in Table 8, among these models, RoBERTa consistently achieved the highest overall performance. Specifically, RoBERTa recorded the best average F1 score (85.55%) and accuracy (85.01%), clearly surpassing other models. DistilBERT, on the other hand, performed poorest, with the lowest average F1 score (81.70%) and accuracy (81.09%). ALBERT and XLNet showed moderate performance, while BERT exhibited intermediate results with slightly better performance than ALBERT and DistilBERT.

Regarding the detection speed (Table 9), DistilBERT was the fastest, averaging 0.016 s per instance. Although RoBERTa did not achieve the fastest detection speed, its average inference time of 0.023 s per instance.

Considering both performance and computational efficiency, the optimized RoBERTa model—with a sequence length of 512, learning rate of 1e-5, warm-up proportion of 0.1, batch size of 32, and trained for 5 epochs—was selected as the final model. This choice was based primarily on its superior accuracy and F1 score, coupled with a sufficiently fast inference time suitable for real-world deployment scenarios.

**Table 9:** Detection speed by BERT based methods.

Method	Seconds per instance						
	Mean	Std	Min	25%	50%	75%	Max
BERT	0.0221	0.0006	0.0215	0.0218	0.0221	0.0225	0.0228
RoBERTa	0.023	0.0006	0.0222	0.0226	0.0229	0.0233	0.0236
ALBERT	0.0197	0.0006	0.019	0.0193	0.0197	0.0201	0.0204
DistilBERT	0.016	0.0005	0.0155	0.0157	0.016	0.0163	0.0166
XLNet	0.0267	0.0006	0.0258	0.0263	0.0267	0.0272	0.0276

### Model evaluation in test data

#### Test data performance

**Fig. 11:**
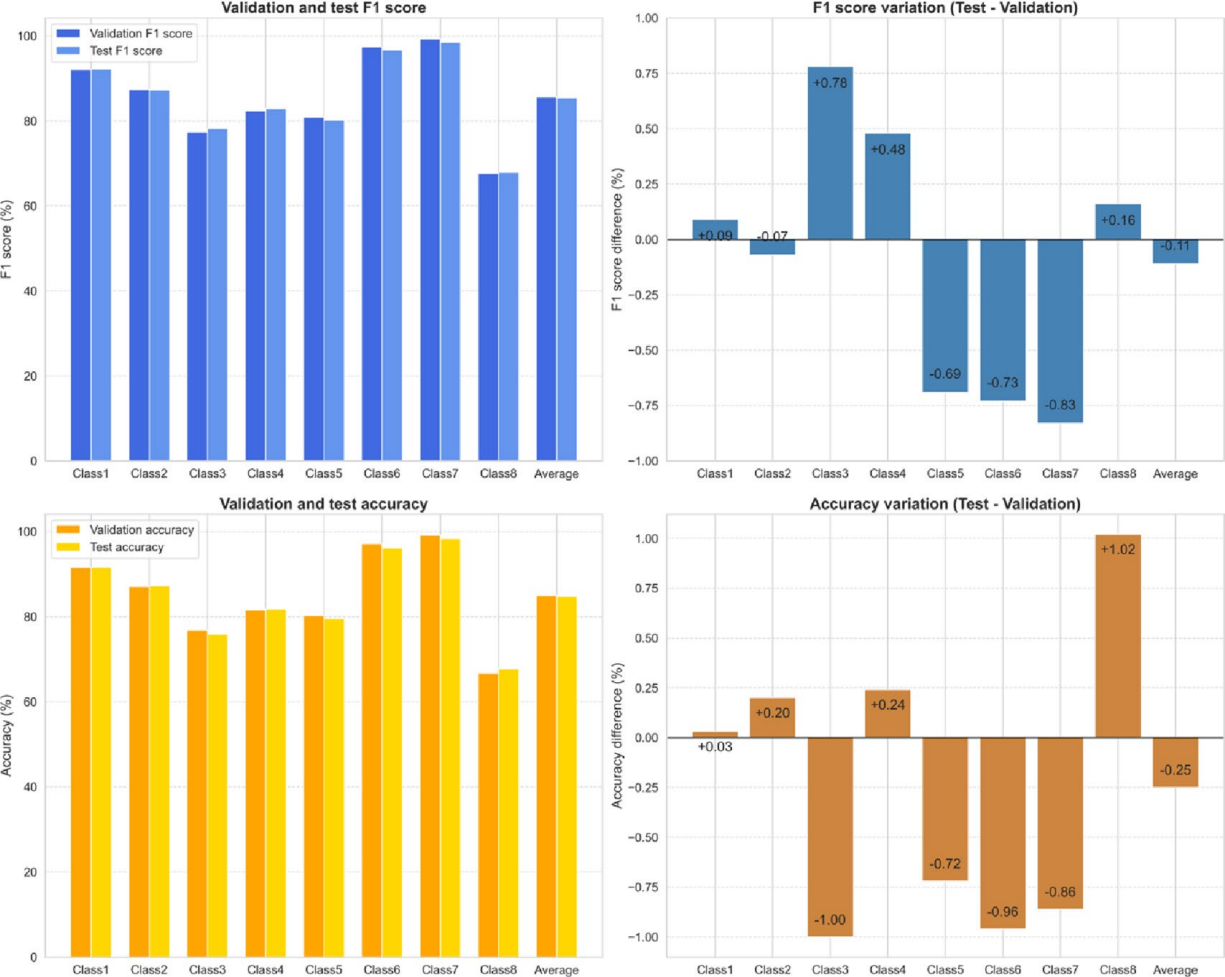
Validation and test results of the best-performing model (RoBERTa-based method).

Figure 11 illustrates RoBERTa’s validation and test performance across different classes. RoBERTa demonstrated robust and consistent predictive performance, with validation F1 scores ranging from 67.65% (Class8) to 99.26% (Class7), averaging at 85.55%, and validation accuracies ranging from 66.72% (Class8) to 99.13% (Class7), averaging 85.01%.

When assessing generalization on the test dataset, RoBERTa showed minimal variation, with F1 scores fluctuating slightly (between − 0.73% and + 0.78%) and accuracies varying between − 1.00% and + 1.02%. This indicates stable and reliable generalization to unseen data, reflecting neither significant overfitting nor underfitting.

**Fig. 12:**
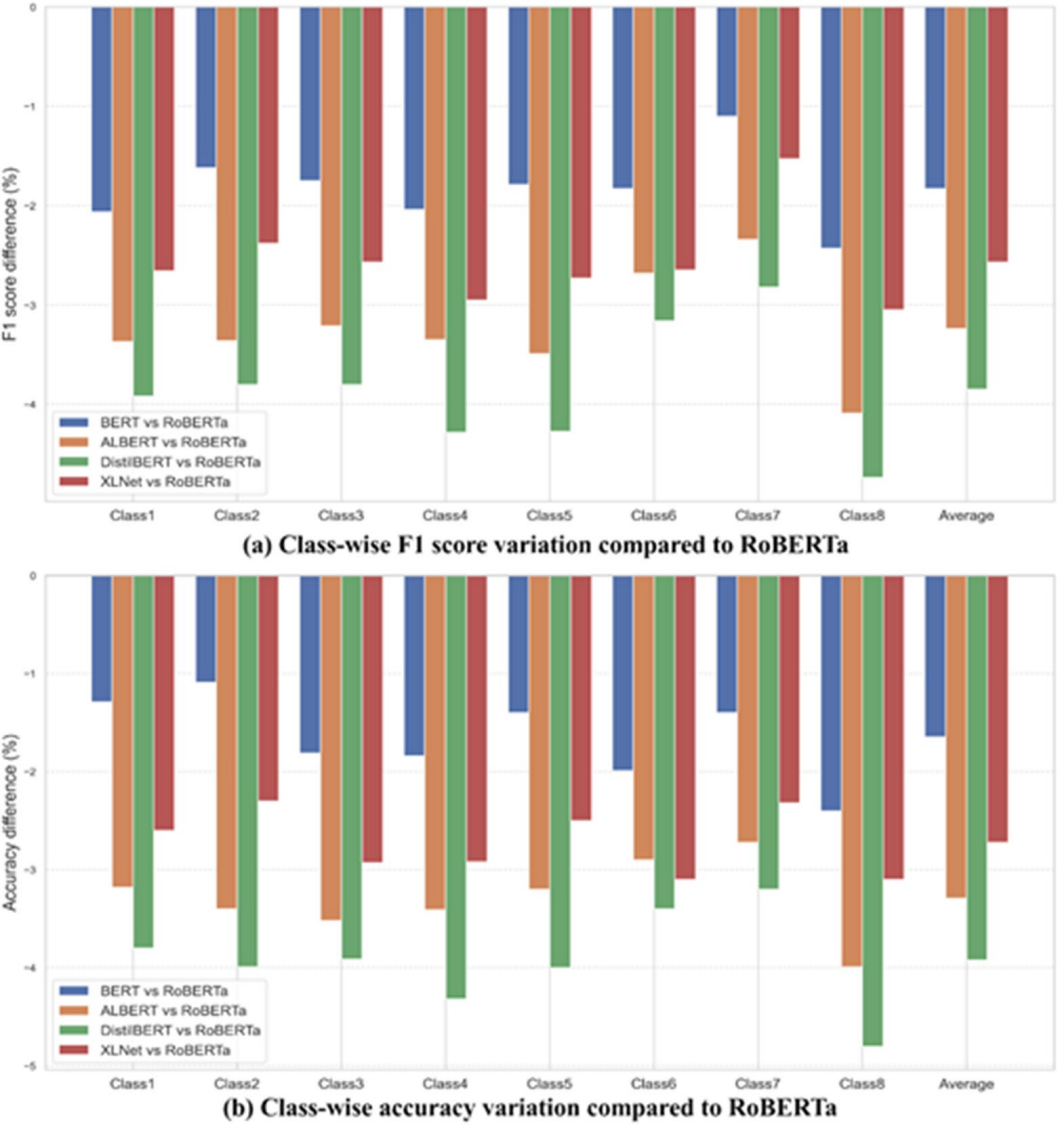
Comparison results of best model with other models.

Figure 12 demonstrates the relative performance of transformer-based models (BERT, ALBERT, DistilBERT, XLNet) compared to RoBERTa as a baseline. Overall, RoBERTa exhibited superior predictive capability, consistently outperforming other models. Specifically, DistilBERT displayed the largest negative variations (up to −4.74% in F1 score and − 4.80% in accuracy), while BERT and XLNet showed moderate negative differences. ALBERT also lagged behind RoBERTa but maintained relatively smaller and more consistent negative differences.

#### Comparison with other detectors

##### Selection of other detectors

To demonstrate the superiority of the proposed RoBERTa-based approach, comparisons were conducted with several widely used text classification methods, including ANN, SVM, DT, LR, 1D CNN, and LSTM. Additionally, various vectorization techniques such as Term Frequency-Inverse Document Frequency (TF-IDF), Bag-of-Words (BOW), 3-gram, Word Embedding, Word2Vec, and FastText were utilized. Hyperparameters for each method were empirically optimized through preliminary experimentation, resulting in a total of 535 evaluated models across all methods. Table 10 summarizes the classification methods along with their optimized hyperparameters and presents the best-performing configurations identified from the test dataset.

**Table 10:** Comparison methods with hyperparameters.

Algorithm	Vectorization method	Hyperparameter	Numbers of models	Best model
ANN	TF-IDF, BOW, 3-gram	Hidden layers: [1,2,3]; Nodes per layer: [64,128,256]; Activation: [relu, tanh]; Learning rate: [0.001,0.01,0.1]	54	TF-IDF, Hidden layers: 2, Nodes: 128, Activation: relu, Learning rate: 0.01
SVM		C: [0.1,1,10,100]; Kernel: [linear, rbf]; Gamma: [scale, auto]	16	TF-IDF, C: 10, Kernel: rbf, Gamma: scale
DT		Max depth: [10,20,30,None]; Criterion: [gini, entropy]; Max features: [auto, sqrt, log2,None]	32	BOW, Max depth: None, Criterion: entropy, Max features: sqrt
LR		C: [0.1,1,5,10,20]; Penalty: [l2]; Solver: [newton-cg, lbfgs, liblinear, sag, saga]	25	TF-IDF, C: 10, Penalty: l2, Solver: saga
1D CNN	Word embedding, Word2Vec, FastText	Conv layers: [1,2]; Filters: [128,256,512]; Kernel sizes: [3,5]; Embedding size: [100,200]; Dropout: [0.2,0.3]; LR: [0.0001,0.001]; Batch size: [32,64]	192	FastText, Conv layers: 2, Filters: 256, Kernel size: 5, Embedding size: 200, Dropout: 0.2, Learning rate: 0.0001, Batch size: 32
LSTM		Hidden layers: [1,2]; Hidden units: [128,256]; Embedding size: [100,200,300]; Dropout: [0.2,0.3,0.5]; Learning rate: [0.0001,0.001]; Batch size: [32,64,128]	216	Word2Vec, Hidden layers: 1, Hidden units: 256, Embedding size: 300, Dropout: 0.3, Learning rate: 0.0001, Batch size: 64

##### Comparison results with other detectors

Tables 11 and 12 compare the performance of the proposed RoBERTa-based method with other machine learning and deep learning models on the test dataset. Table 11 illustrates that the proposed method the RoBERTa-based classifier achieved the highest overall performance on the test set, with an average F1-score of 85.44% and accuracy of 84.76% across eight classes. Relative to the strongest traditional machine-learning baselines—SVM (F1-score = 79.15%) and RF (accuracy = 78.73%)—RoBERTa yields absolute gains of + 6.29% points in F1-score and + 6.03% points in accuracy. Compared with the best non-transformer deep-learning model (LSTM; F1-score = 80.89%, accuracy = 79.65%), the improvements are + 4.55 and + 5.11% points, respectively, with the largest margins in the more challenging categories (Classes 3 and 8).

Additionally, Table 12 highlights the detection speed advantage of the RoBERTa-based method, achieving an average inference time of 0.0221 s per instance, significantly faster than other evaluated models. Despite having more parameters, the superior detection speed of the RoBERTa-based method can be primarily attributed to its transformer-based architecture, optimized embedding techniques, and effective GPU acceleration, enabling efficient parallel processing. In contrast, traditional machine learning models and certain deep learning architectures typically rely on CPU-based computations, which restricts their inference speed.

**Table 11:** Comparison results of best model on test set.

Category	Class1	Class2	Class3	Class4	Class5	Class6	Class7	Class8	Average
Proposed method									
F1 score	92.13	87.29	78.17	82.89	80.17	96.66	98.43	67.81	85.44
Accuracy	91.55	87.22	75.84	81.78	79.49	96.16	98.27	67.74	84.76
ANN									
F1 score	86.94	82.51	44.38	79.7	78.97	95.46	97.67	63.41	78.63
Accuracy	86.34	83.53	45.25	76.92	75.24	94.7	96.95	66.41	78.17
SVM									
F1 score	87.26	84.43	45.73	81.08	76.92	95.53	96.62	65.66	79.15
Accuracy	86.81	83.19	44.44	78.97	76.32	95.45	95.04	66.47	78.34
DT									
F1 score	81.34	82.52	43.32	78.75	78.3	95.72	94.85	65.33	77.52
Accuracy	80.5	84.49	45.19	77.19	77.83	92.68	96.37	64.9	77.39
RF									
F1 score	89.17	85.96	43.31	78.9	75.44	92.13	95.24	63.16	77.91
Accuracy	87.65	85.84	45.14	79.82	77.24	94.44	94.04	65.63	78.73
LR									
F1 score	87.37	84.35	47.04	78.78	79.33	91.72	94.45	66.42	78.68
Accuracy	88.03	83.05	42.16	78.02	75.52	91.33	94.16	66.72	77.37
1D CNN									
F1 score	89.75	83.99	56.18	82.1	78.27	94.7	94.65	64.44	80.51
Accuracy	89.06	84.6	54.8	78.07	75.57	93.13	94.3	65.02	79.32
LSTM									
F1 score	89.28	84.87	57.56	81.9	79.53	92.42	96.52	65.02	80.89
Accuracy	86.97	85.61	53.49	77.88	77.96	92.31	96.47	66.51	79.65

**Table 12:** Detection speed by each method (seconds per instance).

Method	Mean	Std	Min	25%	50%	75%	Max
Proposed method	0.0221	0.0006	0.0215	0.0218	0.0221	0.0225	0.0228
ANN	0.0274	0.0022	0.0259	0.0264	0.0274	0.0286	0.0294
SVM	0.0259	0.0009	0.0242	0.0254	0.0259	0.0263	0.0266
DT	0.0292	0.0021	0.0269	0.0282	0.0292	0.0303	0.0303
RF	0.0314	0.0014	0.0301	0.0307	0.0314	0.0321	0.0328
LR	0.0269	0.0015	0.0258	0.0262	0.0269	0.0277	0.0283
1D CNN	0.0293	0.0011	0.0279	0.0288	0.0293	0.0299	0.0308
LSTM	0.0281	0.002	0.0265	0.0271	0.0281	0.0291	0.0292

### SHAP analysis

**Fig. 13:**
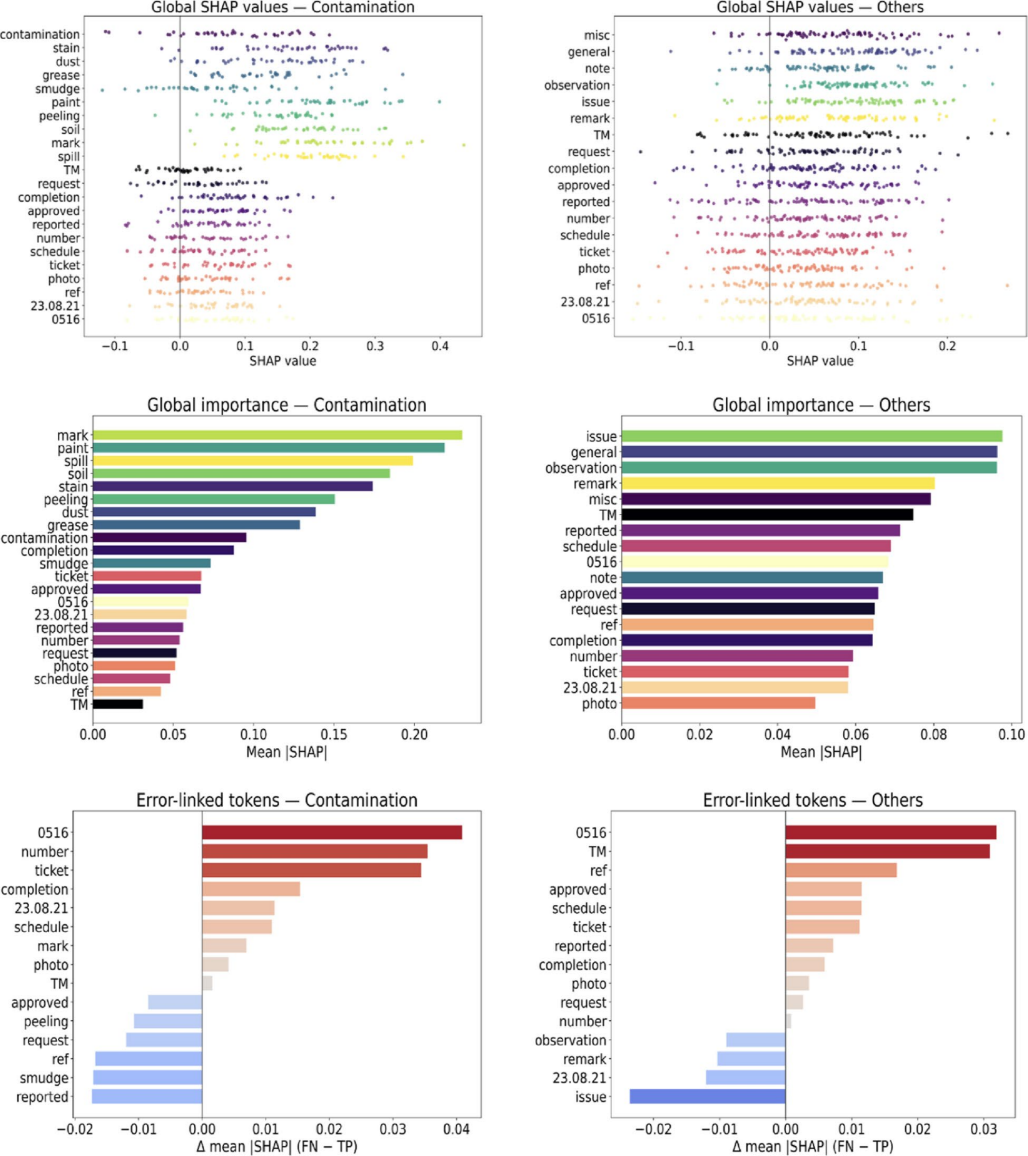
Results of SHAP analysis.

Figure 13 summarizes SHapley Additive exPlanations (SHAP)-based behavior for “Contamination” and “Others”—the two classes with the lowest F1 score and accuracy in both the validation and test sets. For “Contamination”, global SHAP indicates strong reliance on defect cues: mean |SHAP| for oil (~ 0.22), stain (~ 0.20), dust (~ 0.17), and gap/closer/gasket (~ 0.16–0.19) is roughly 3–4× higher than for administrative terms (TM/request/completion ~ 0.03–0.06). Δ(FN − TP) identifies administrative markers as error drivers: request (+ 0.06–0.09), completion (+ 0.07–0.10), TM (+ 0.04–0.08), and date/ID codes (+ 0.04–0.07) gain attribution in false negatives, diluting contamination cues.

For “Others”, the pattern reverses: administrative/generic tokens dominate (mean |SHAP| for request/completion/ticket/photo ~ 0.05–0.07), while class-generic words (misc/note/observation) are weaker (~ 0.03–0.04). Δ(FN − TP) again flags administrative terms as principal error sources (+ 0.05–0.09), consistent with this class’s catch-all nature.

Notably, a small set of administrative tokens accounts for substantial misclassification pressure in both classes (attribution shifts of + 0.04 to + 0.10), motivating abbreviation/administrative-text normalization, refined “Others” labeling, and negation-aware preprocessing to improve class- specific F1 score.

### Potential applications of research findings

The proposed transformer-based classifiers—particularly RoBERTa—can automate triage of fire-door defect reports in computer-aided maintenance systems, enabling rapid prioritization of safety-critical repairs and efficient work-order bundling. Integrated with digital-twin, and Building Information Modeling (BIM) environments, they can surface defect locations, track remediation, and support audit readiness for regulatory compliance^55,56^. Near-real-time inference (~ 0.02–0.03 s per instance) enables monitoring dashboards and contractor-performance benchmarking. Beyond fire doors, the workflow extends to other textual quality-assurance records and multilingual logs, facilitating preventive-maintenance planning and evidence-based resource allocation. Moreover, the outputs can support cost-effectiveness and Return-On-Investment (ROI) analyses by quantifying accuracy-driven reductions in reinspection effort, response time, and safety risk^57–60^.

## Conclusions

This study proposed and evaluated five transformer-based models (BERT, RoBERTa, ALBERT, DistilBERT, and XLNet) for detecting fire door defects, utilizing optimized hyperparameters such as sequence length, epochs, and batch size. In total, 1,458 model variants were developed and evaluated. The dataset comprised 4,212 real-world fire door defect reports collected from apartment complexes, covering 8,786 household units. Eight defect categories were identified, including seven common defects and one minor defect.

Among the evaluated methods, the optimized RoBERTa model demonstrated the highest performance. Specifically, on the test dataset, RoBERTa achieved the following F1 scores per defect category: frame gap (92.13%), door closer adjustment (87.29%), contamination (78.17%), dent (82.89%), scratch (80.17%), sealing components (96.66%), mechanical operation components (98.43%), and others (67.81%), resulting in an average F1 score of 85.44%. Regarding accuracy, RoBERTa attained the following results: frame gap (91.55%), door closer adjustment (87.22%), contamination (75.84%), dent (81.78%), scratch (79.49%), sealing components (96.16%), mechanical operation components (98.27%), and others (67.74%), with an overall average accuracy of 84.76%.

Furthermore, the RoBERTa-based model significantly outperformed six conventional classifiers— ANN, SVM, DT, LR, 1D CNN, and LSTM—across 535 tuned variants. On the test set it achieved an average F1-score of 85.44% and accuracy of 84.76%, exceeding the best traditional baseline (SVM: F1-score 79.15%; Random Forest: accuracy 78.73%) by + 6.29 and + 6.03% points, respectively. Relative to the strongest non-transformer deep-learning baseline (LSTM: F1-score 80.89%, accuracy 79.65%), the improvements are + 4.55 and + 5.11% points, underscoring the method’s robustness and practical value.

Despite promising results, several limitations warrant further study. The corpus was assembled from a small number of companies within a fixed period, and inspector training levels varied; consequently, writing style and label quality may not reflect broader practice. As with any data-driven approach, performance depends on the quality and representativeness of the training texts, so external validity will hinge on how closely new inspection narratives resemble those used here. The study also did not quantify economic benefit; life-cycle cost and ROI analyses tailored to this triage workflow and to alternative deployment contexts remain outstanding.

Future work should expand evaluation to multi-source datasets spanning additional contractors, time windows, and inspector backgrounds to strengthen generalizability. It should also assess cross-lingual robustness by training and testing on the original Korean texts and other languages, using multilingual encoders, back-translation consistency checks, and lightweight adapters where appropriate. Finally, the impact of translation should be examined systematically by comparing human and machine translations and measuring their effects on class-wise performance and error patterns. These steps will clarify how domain shift, language choice, and translation quality influence reliability and guide practical deployment at scale.

## Data Availability

The datasets generated during and/or analyzed during the current study are available from the corresponding author on reasonable request.
